# Macrophage metabolism, phenotype, function, and therapy in hepatocellular carcinoma (HCC)

**DOI:** 10.1186/s12967-023-04716-0

**Published:** 2023-11-15

**Authors:** Jingquan Huang, Qiulin Wu, David A. Geller, Yihe Yan

**Affiliations:** 1 Department of General Surgery, The Second Affiliated Hospital of Guangxi Medical University, Nanning, 530007 Guangxi China; 2 Department of Surgery, Thomas E. Starzl Transplantation Institute, University of Pittsburgh Medical Center, Pittsburgh, PA 15260 USA

**Keywords:** Macrophage, Metabolism, Phenotype, Function, Tumor microenvironment, Hepatocellular carcinoma

## Abstract

The pivotal role of the tumor microenvironment (TME) in the initiation and advancement of hepatocellular carcinoma (HCC) is widely acknowledged, as it fosters the proliferation and metastasis of HCC cells. Within the intricate TME of HCC, tumor-associated macrophages (TAMs) represent a significant constituent of non-malignant cells. TAMs engage in direct communication with cancer cells in HCC, while also exerting influence on other immune cells to adopt a tumor-supportive phenotype that facilitates tumor progression. Among the multifaceted mechanisms at play, the metabolic reprogramming of both tumor cells and macrophages leads to phenotypic alterations and functional modifications in macrophages. This comprehensive review elucidates the intricate interplay between cellular metabolism and macrophage phenotype/polarization, while also providing an overview of the associated signaling molecules and potential therapeutic strategies for HCC.

## Introduction

Hepatocellular carcinoma (HCC) is a prevalent malignancy with significant global mortality rates. The etiology and progression of HCC are influenced by various factors, such as hepatitis B virus (HBV) and HCV infection, aflatoxin exposure, alcohol consumption, non-alcoholic fatty liver disease (NAFLD), non-alcoholic steatohepatitis (NASH), and cirrhosis. While surgical resection, ablation, and liver transplantation are currently the most efficacious treatments for early-stage HCC, the diagnosis of HCC often occurs at an advanced stage, resulting in poor prognosis [1–3]. The advent of systemic therapeutics for advanced HCC, including immune checkpoint inhibitors (ICIs) [4, 5], tyrosine kinase inhibitors (TKIs) [6–8], and anti-angiogenic antibodies [9, 10], has improved patient outcomes by demonstrating some anti-tumor effects [1, 2, 11]. However, the efficacy of these therapeutics is hindered by primary and secondary resistance, posing a significant challenge [12].

The tumor microenvironment (TME) plays a critical role in modulating the response to anti-tumor therapy in HCC. By orchestrating alterations in the surrounding milieu of HCC cells, including endothelial cells, stromal cells, immune cells, and associated cytokines, a pro-tumor TME is established, thereby facilitating HCC cell proliferation and impeding the efficacy of anti-tumor interventions [13–15]. Additionally, extracellular metabolites within the TME serve as both energy sources and mediators of intercellular communication. Notably, tumor cells exploit these metabolites to subvert immune cells, redirecting their function from anti-tumor to pro-tumor activities [16]. Concurrently, non-malignant cells contribute to tumor support by releasing metabolites into the TME [17].

Macrophages constitute a significant proportion of immune cells and exert crucial functions within the TME of HCC. Notably, the intrinsic characteristics of tumor cells and extrinsic environmental factors induce alterations in the metabolism, phenotype, and biological functions of macrophages [18–20], thereby enabling their reprogramming to support tumor growth. Of particular importance, changes in cellular metabolism can profoundly impact the functional properties of macrophages [21–23]. Consequently, it is imperative to investigate the intricate interplay between macrophage metabolism, phenotype, and function. In this comprehensive review, we present the latest advancements in understanding macrophage phenotypes, functions, and metabolism, while also exploring potential therapeutic strategies for HCC that target key molecules within these immune cells.

## Phenotype and function of macrophages

Macrophages, as a crucial component of the innate immune system, are ubiquitously distributed in the bloodstream and various tissues throughout the body. Their significance extends to autoimmune disorders and malignancies, where they play a pivotal role in processes such as angiogenesis and tumor progression. Originating from monocytes in the peripheral blood, macrophages undergo differentiation into distinct functional subpopulations upon local recruitment and stimulation by specific chemokines [24]. Additionally, a subset of macrophages arises from tissue-resident macrophages. These versatile immune cells exhibit a diverse range of functions, including cellular debris clearance, promotion of angiogenesis for tissue repair, cytokine release, phagocytosis-mediated inflammatory responses, and tumor cell elimination [25–28]. Meanwhile, macrophages possess a high degree of plasticity and can adapt to environmental changes by modulating their cellular metabolism and functional phenotype [29].

Macrophages have been classified into two primary categories based on their activation status and biological functions: classically activated macrophages (M1-like phenotype) and alternatively activated macrophages (M2-like phenotype) [30]. The differentiation of these macrophage subtypes occurs under distinct conditions. While recent advancements in single-cell RNA sequencing techniques have revealed the existence of additional macrophage phenotypes, the M1 and M2 categories remain the predominant phenotypes used to distinguish macrophage populations.

### M1-like phenotype macrophages

**Fig. 1:**
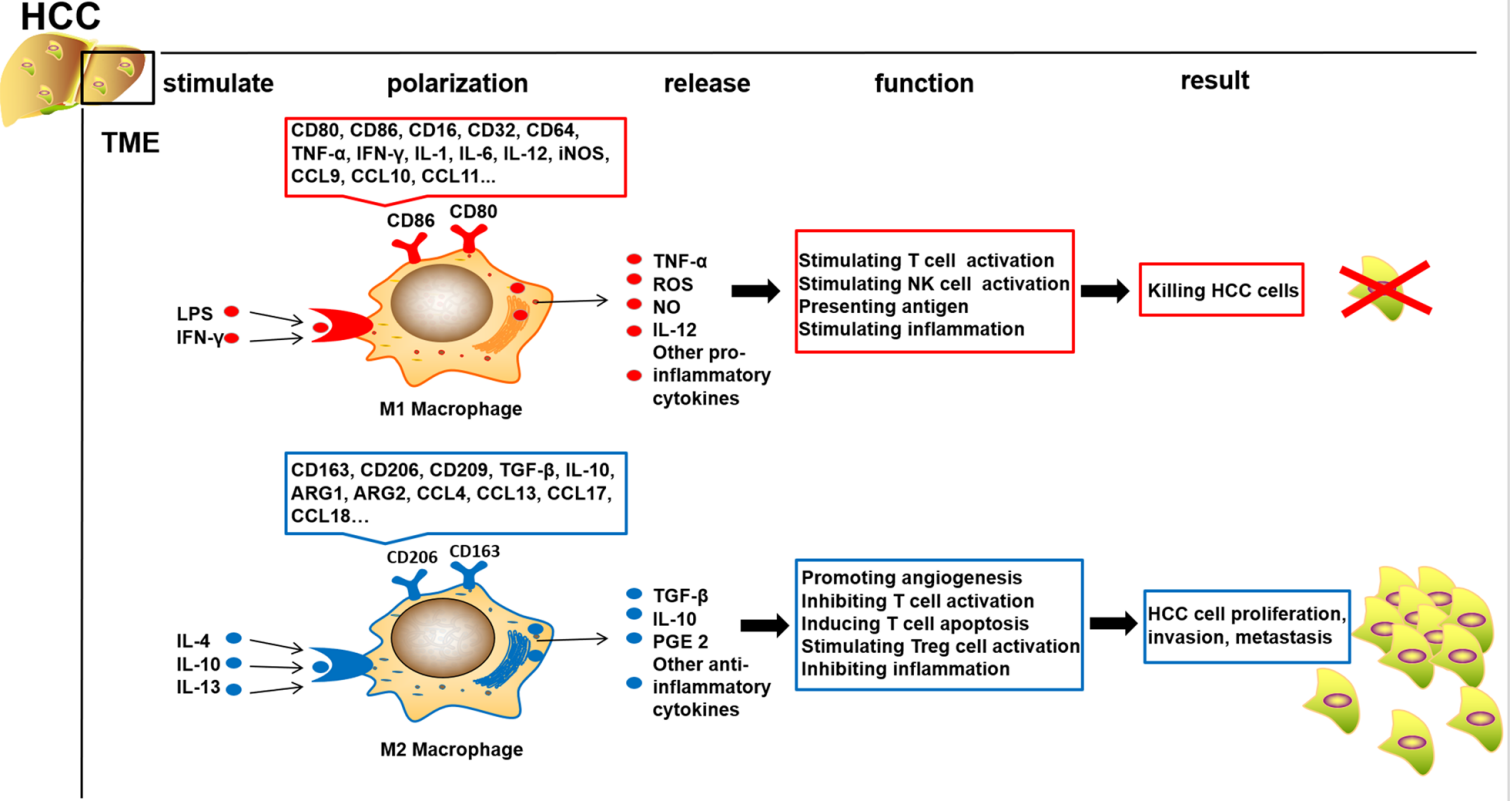
The differentiation of macrophages into distinct phenotypes, namely M1 and M2-like, is influenced by various stimulators. For instance, the exposure to LPS and IFN-γ induces macrophages to adopt an M1-like phenotype. M1-like macrophages express specific markers such as CD80, CD86, CD16, CD32, CD64, among others. These M1-like macrophages are capable of secreting pro-inflammatory factors including TNF-α, NO, IL-12, among others. Furthermore, they can activate T cells and NK cells, thereby mediating anti-tumor immunity against HCC cells. Conversely, the presence of IL-4, IL-10, and IL-13 can induce macrophages to undergo M2-like polarization. M2-like macrophages express specific markers such as CD163, CD206, CD209, TGF-β, IL-10, among others. These M2-like macrophages play a role in promoting angiogenesis, inhibiting T cell activation, and inducing T cell apoptosis, thereby assisting HCC cells in evading immune surveillance

Stimulation with cytokines such as lipopolysaccharide (LPS), Toll-like receptor (TLR) ligands, and interferon-γ (IFN-γ) elicits the induction of a pro-inflammatory M1-like phenotype in macrophages. These M1-like macrophages exhibit heightened expression of T-lymphocyte activating antigens (CD80 and CD86) and possess a multitude of capabilities, including the secretion of pro-inflammatory cytokines, generation of reactive oxygen species (ROS), expression of inducible nitric oxide synthase (iNOS) and subsequent production of nitric oxide (NO), as well as the synthesis of interleukin 12 (IL-12) to activate other immune cells. This activation cascade ultimately enhances the antigen-presenting capacity of macrophages, facilitating the activation of cytotoxic T lymphocytes and promoting the eradication of tumor cells [25, 30–32] (Fig. 1).

Bufalin has demonstrated its ability to modulate anti-tumor immunity by inhibiting the expression of nuclear factor-κB (NF-κB) p50, leading to an increase in p65–p50 heterodimerization. This subsequently activates NF-κB signaling, resulting in the production of immune stimulatory factors. These factors induce the reprogramming of M2-like macrophages to M1-like phenotypes, thereby facilitating T cell-associated anti-tumor immunity [33]. Additionally, the matricellular protein SPON2 acts as a suppressor of HCC by activating RhoA and Rac1 through the integrin SPON2-α5β1. This activation promotes F-actin recombination, which in turn facilitates the infiltration of M1-like macrophages [34]. Furthermore, forkhead box protein O1 (FOXO1), derived from HCC, not only targets tumor cells but also synchronizes with re-educated macrophages. This synchronization is partially dependent on FOXO1 transcriptionally modulating the interferon regulatory factor 1 (IRF1)/nitric oxide (NO) axis in macrophages, resulting in the suppression of HCC cells [35].

### M2-like phenotype macrophages

Conversely, the cytokines IL-4, IL-10, and IL-13 have been shown to drive the polarization of macrophages towards an M2-like phenotype with anti-inflammatory characteristics. M2-like macrophages are characterized by high expression of scavenger receptors (CD163) and mannose receptors (CD206) (Fig. 1). These macrophages secrete anti-inflammatory cytokines that play a crucial role in suppressing immune responses, regulating hypoxia, promoting angiogenesis, facilitating tumor invasion, metastasis, and progression, as well as aiding tumor cells in evading anti-tumor immune responses [21, 36–40].

The PKC/ZFP64/CSF1 axis within the HCC-regulated TME is characterized by its ability to induce polarization of M2-like macrophages and mediate immunosuppression, resulting in resistance to anti-PD1 therapy. Mechanistically, PKC phosphorylates ZFP64, leading to its nuclear translocation and subsequent activation of CSF1 at the transcriptional level. This activation of CSF1 transforms macrophages into M2-like phenotypes. Consequently, the protein kinase inhibitor Gö6976 and the multiple kinase inhibitor lenvatinib are able to disrupt this axis, thereby reversing the immunosuppressive TME and reprogramming the macrophage phenotype to increase susceptibility to anti-PD1 therapy [41]. Additionally, the IRF2/β-catenin pathway plays a regulatory role in lenvatinib resistance in HCC cells. Targeting this pathway may hold promise in improving the therapeutic efficacy of lenvatinib for HCC treatment [42].

Meanwhile, the polarization of macrophages towards an M2-like phenotype was observed in the presence of TREM1 expression. Notably, the down-regulation of TREM1 resulted in the transformation of M2-like macrophages into an M1-like phenotype, thereby exerting a preventive effect on the invasion and metastasis of HCC cells. This transformation was achieved through the suppression of the PI3K/AKT/mTOR signaling pathway [43].

## Communication of TAMs with other immune cells in HCC

Macrophages present within the TME are commonly referred to as TAMs, constituting the predominant immune cell population infiltrating various tumor types. TAMs typically exhibit M2-like phenotypes, characterized by their secretion of anti-inflammatory cytokines and angiogenetic factors, thereby creating an inflammatory milieu that supports cancer cell survival. Concurrently, TAMs facilitate angiogenesis and tumor cell proliferation, while also bolstering tumor cell resistance to chemotherapy agents, including cytotoxic agents and checkpoint inhibitors. Additionally, TAMs play a role in suppressing anti-tumor immune responses. These multifaceted functions are often mediated through the cross-talk between TAMs and other immune cells [44–46].

### Communication of M2-like TAMs with other immune cells in HCC

**Fig. 2:**
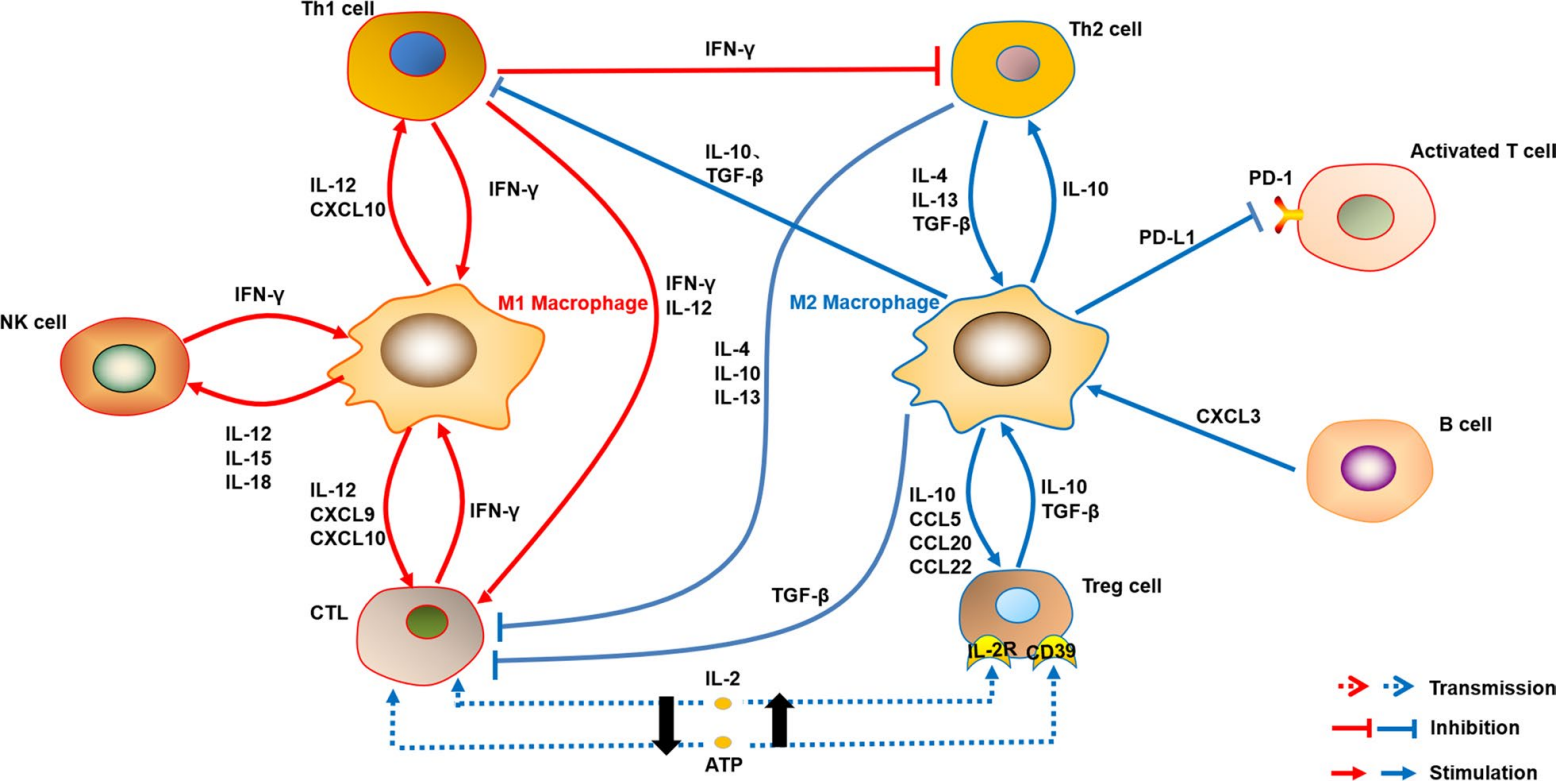
Communication of macrophages with other immune cells. M1-like macrophages and M2-like macrophages possess different effects on other immune cells in the TME, respectively. Moreover, the other immune cells regulate macrophage polarization through their specific ways

TAMs exhibit close interactions with other immune cells within the TME. Notably, TAMs have the ability to dampen the anti-tumor immune responses mediated by M1-like macrophages [47]. Additionally, TAMs secrete IL-10, which serves to suppress the function of Th1 cells while promoting the growth and development of Th2 cells [48]. Th2 cells, in turn, produce IL-4, thereby facilitating the development of TAMs. Functionally, Th2 cells exhibit characteristics that are opposite to Th1 cells, as they actively suppress the activation of cytotoxic T lymphocytes (CTLs). Simultaneously, the IL-10 produced by TAMs activates regulatory T (Treg) cells, leading to the inhibition of macrophage response to LPS and consequently suppressing the immune response, thereby promoting tumor progression. Furthermore, TAMs produce transforming growth factor-beta (TGF-β), which hinders the development of Th1 cells and CTLs, subsequently diminishing their anti-tumor activities [49] (Fig. 2).

Furthermore, TAMs exhibit high expression of the CD48 protein, which interacts with the 2B4 receptor (CD244) present on natural killer (NK) cells. This interaction ultimately leads to the rapid activation, exhaustion, and subsequent demise of NK cells [50]. Simultaneously, TAMs contribute to tumor progression by releasing anti-inflammatory cytokines, such as prostaglandin E2 (PGE2), and expressing programmed death ligand 1 (PD-L1) on their surface. PD-L1 engages with the PD-1 receptor on activated T cells, inducing apoptosis and thereby impeding immune responses [51, 52] (Fig. 2).

### Communication of M1-like TAMs with other immune cells in HCC

In addition to the presence of M2-like TAMs, the TAM population also includes M1-like macrophages, which exert distinct effects on immune cells. Unlike M2-like macrophages, M1-like TAMs play a crucial role in promoting anti-tumor responses by activating NK cells, Th1 cells, and CTL. Additionally, they secrete IL-15 and IL-18 to stimulate NK cells [53]. The activated NK cells, in turn, produce IFN-γ, which induces macrophages to polarize towards the M1 phenotype [54]. Furthermore, various immune cells, including CD8 T cells, specific subsets of CD4 T cells, and γδ T cells, are capable of producing IFN-γ, thereby promoting M1-like polarization [55]. Thus, this reciprocal feedback loop contributes to the establishment of an anti-tumor immune microenvironment (Fig. 2). In light of these findings, inhibiting M2-like TAM polarization may represent a promising strategy for HCC immunotherapy.

## TAMs and associated clinical application in HCC

### The characteristic of TAMs

As previously mentioned, TAMs do not solely exhibit the M2-like phenotype. Extensive investigations into macrophages in human cancers have revealed that TAMs express markers associated with both M1-like and M2-like phenotypes, indicating a mixed population [23, 56–58]. Since M1-like and M2-like macrophages exert pro-inflammatory and anti-inflammatory effects, respectively, the phenotypes and functions of TAMs can undergo transformation under various intrinsic and extrinsic conditions [52, 59, 60].

During tumorigenesis, M2-like macrophages can undergo conversion to the M1-like phenotype, enabling them to combat tumor cells. Conversely, tumors have the ability to induce the conversion of the M1-like phenotype to the M2-like phenotype, thereby shielding themselves from immune cell-mediated damage [61–63]. Hence, the different subpopulations of macrophages can undergo interconversion rather than maturation and differentiation into distinct subpopulations. Moreover, a high density of TAMs in the tumor microenvironment has been correlated with a poor prognosis in patients, further confirming the tumor-promoting role of TAMs [51, 64, 65]. Consequently, targeting TAMs may represent a viable approach for tumor immunotherapy [66, 67].

### TAMs-associated therapy in HCC

Targeting TAMs for tumor immunotherapy has been explored in clinical trials for the treatment of HCC. In one such trial, the combination of a phosphatidylserine (PS) targeting agent (2aG4) with sorafenib has demonstrated increased apoptosis of tumor cells and elevated levels of M1-like macrophages, while simultaneously reducing tumor microvasculature genesis and density of M2-like macrophages. This ongoing Phase I clinical study focuses on HCC patients and holds great promise as a therapeutic target [68].

Furthermore, the combination of tyrosine kinase inhibitors (TKIs) with immune checkpoint inhibitors (ICIs) has shown potential in enhancing anti-tumor immunity and inhibiting M2-like polarization. For instance, regorafenib has been found to enhance M1-like TAM polarization and increase the M1/M2 ratio. When combined with an ICI, regorafenib exhibits enhanced anti-HCC effects [69, 70]. Additionally, the compound kushen injection (CKI) has been shown to activate the TNFR1/NF-κB/p38/MAPK pathway, thereby reducing the immunosuppressive effects of TAMs and promoting T cell-associated cytotoxicity, leading to apoptosis of HCC cells. CKI also improves the efficacy of sorafenib, and the combination treatment demonstrates stronger anti-HCC responses [71].

Additionally, the activation of the Wnt2b/β-linked c-Myc signaling pathway has been found to promote the conversion of M2-like TAMs, thereby enhancing the progression of HCC. Conversely, the down-regulation of Wnt2b/β-linked protein expression through the use of Toll-like receptor 9 (TLR9) agonist CpG ODN has been shown to inhibit M2-like TAM polarization and exert anti-tumor effects [72]. Moreover, the long-stranded noncoding RNA (lncRNA) known as miR4458HG, derived from tumor cells and encapsulated in exosomes, has been identified as an oncogenic factor that promotes the conversion of TAMs into an M2-like phenotype. This conversion is achieved through the upregulation of arginase 1 (ARG1) expression, creating a microenvironment conducive to HCC progression [73]. Also, the Nogo-B-Yap/Taz axis has been implicated in the polarization of M2-like TAMs, leading to a tumor-promoting effect and worse prognosis in HCC patients. Knockdown of Nogo-B or the use of Verteporfin, an inhibitor of Yap, has been shown to significantly inhibit the activation of the Nogo-B-Yap/Taz axis-mediated M2-like macrophages, thereby suppressing HCC cell proliferation [74]. Collectively, these pathways present promising therapeutic options for the treatment of HCC.

## Cell metabolism and function

**Fig. 3:**
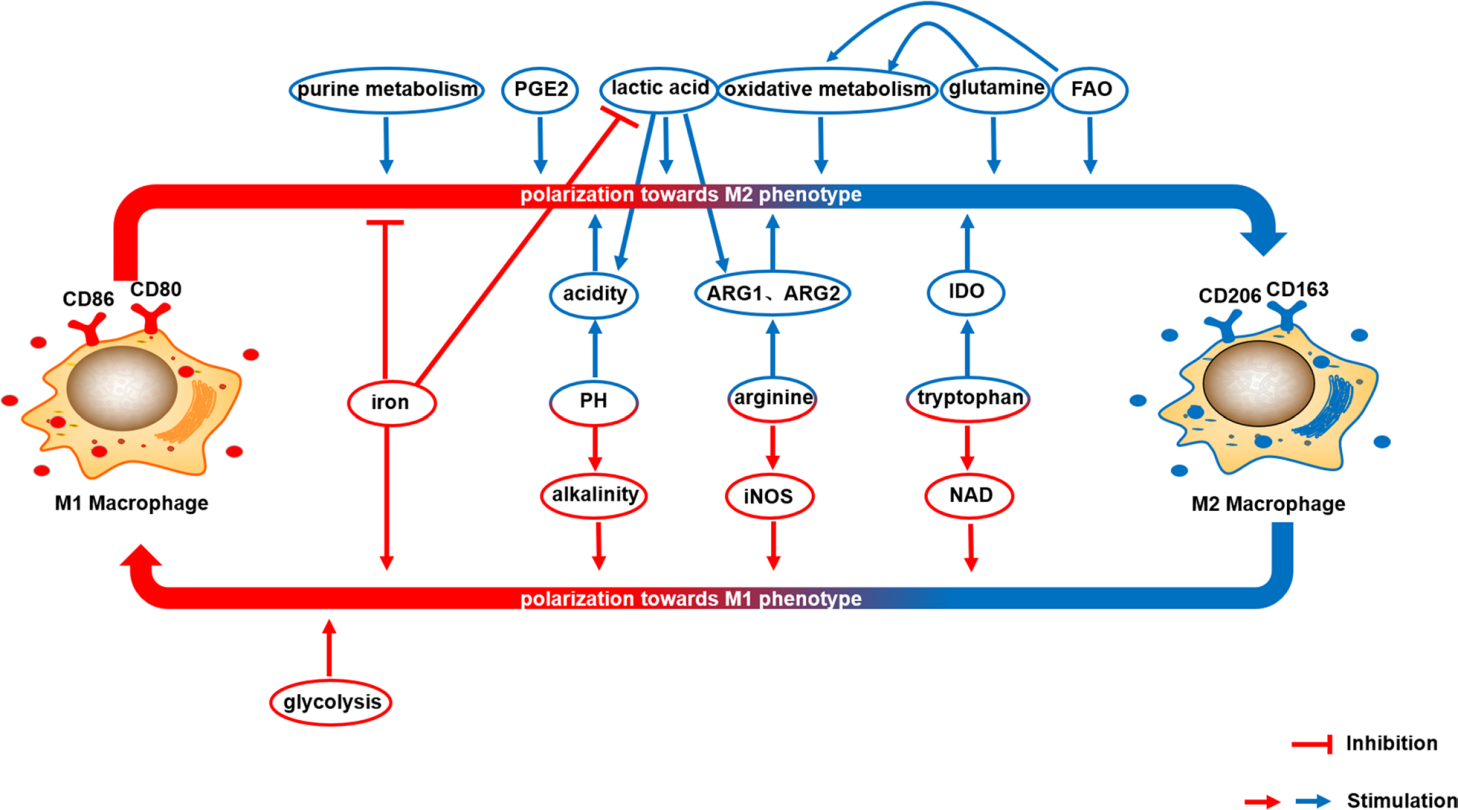
Metabolic processes play a crucial role in regulating the polarization of macrophages. The phenotype of macrophages is intricately linked to their metabolic state. Various physiological pathways involved in metabolism have the ability to influence macrophage function and polarization. Notably, glycolysis, iNOS-associated arginine metabolism, NAD-associated tryptophan metabolism, alkaline environment, and iron uptake have been identified as inducers of M1-like polarization. Conversely, FAO, PGE2 production, oxidative metabolism, lactate production, glutamine-associated metabolism, ARG-associated arginine metabolism, IDO-associated tryptophan metabolism, purine metabolism, and acidic environment have been implicated in the promotion of M2-like polarization. These metabolic pathways and environmental factors contribute to the determination of macrophage polarization and subsequent functional outcomes

Tumors exhibit a strong correlation between cellular metabolism and various cell biological functions. The maintenance of essential cellular functions relies on the availability of energy and nutrients derived from metabolites, such as adenosine 5′-triphosphate (ATP) and various biomolecules like lipids, amino acids, and nucleotides. Numerous studies have extensively explored the impact of metabolism on the biological functions of immune cells, along with the underlying molecular mechanisms involved. Importantly, the metabolic phenotype of macrophages undergoes reprogramming, which significantly influences their biological behavior in HCC (Fig. 3). The physiological pathways associated with metabolism play a crucial role in modulating macrophage function and polarization, and will be comprehensively discussed in the subsequent sections.

### Glucose metabolism

ATP serves as the primary source of cellular energy, without which cells are unable to execute their biological functions. The production of ATP primarily occurs when cells uptake extracellular glucose during the process of catabolism. Two distinct pathways, namely glycolysis and oxidative phosphorylation (OXPHOS), are responsible for the degradation of glucose and subsequent ATP generation. Glycolysis represents a common stage in which organisms undergo glucose catabolism, while OXPHOS serves as the principal mechanism for glucose oxidation, thereby providing energy for organisms [75]. The energy supply for most normal cells heavily relies on ATP derived from OXPHOS.

Upon stimulation by M1-like phenotypic activators such as LPS and IFN-γ, macrophages undergo a transformation in their cellular function accompanied by metabolic alterations. LPS induces polarization towards the M1-like phenotype by activating downstream TLR-induced pathways, leading to the stabilization of hypoxia-inducible factor 1a (HIF1α) expression in macrophages. This, in turn, enhances the activity of mechanistic target of rapamycin (mTOR), promoting glycolytic activity, augmenting the pentose phosphate pathway (PPP), and facilitating de novo synthesis of fatty acids. The significance of glycolysis in M1-like macrophages has been further substantiated by studies demonstrating that inhibitors of glycolysis, such as mTOR inhibitors, can suppress M1-like polarization. Conversely, inhibition of mitochondrial OXPHOS activity and tricarboxylic acid (TCA) cycle by suppressing succinate catabolism in macrophages does not decrease the M1-like phenotype. This suggests that M1-like macrophages do not rely on OXPHOS, but rather their metabolic mode is dependent on glycolysis [76]. Thus, the metabolic characteristics of M1-like macrophages primarily encompass enhanced glycolysis, increased synthesis of fatty acids, and inhibition of the TCA cycle [77, 78].

Glyceraldehyde-3-phosphate dehydrogenase (GAPDH), a crucial enzyme in glycolysis, exhibits significantly decreased activity in M2-like macrophages compared to M1-like macrophages, indicating a reduced reliance on glycolytic metabolism in M2 macrophages [79]. Within the TME, liver cancer cells and macrophages compete for limited glucose resources. To avoid this competition, M2-like macrophages downregulate their own glucose metabolism, leading to an elevated glucose level in the TME. Consequently, cancer cells are able to utilize more glucose for energy supply, thereby promoting their growth. The diminished intracellular glucose metabolism in macrophages facilitates M2-like polarization, which subsequently contributes to tumor progression. Overall, alterations in glucose metabolism play a transformative role in shaping macrophage phenotype and function.

### Lipid metabolism

#### Fatty acid oxidation

In addition to glucose metabolism, lipid catabolism, specifically fatty acid oxidation (FAO), plays a pivotal role in cellular metabolism. Fatty acids (FA) serve as indispensable constituents of cell membrane structure and serve as crucial sources of energy supply. They contribute to various cellular processes including cell membrane composition, homeostasis, and motility. FAO is prominently observed in metabolically active tissues such as cardiac muscle, skeletal muscle, and liver. Under oxygen-rich conditions, FAO generates a greater amount of ATP through the TCA cycle compared to glucose oxidation, thereby serving as a vital energy source for cellular development and function [12, 75, 80]. The accumulation of lipids promotes the proliferation and growth of HCC cells, exacerbating the progression of HCC. Enzymes involved in FAO exhibit tumor-inhibitory effects in HCC. Enhancing β-oxidation can diminish the availability of FA in HCC cells, thereby offering a potential therapeutic approach for HCC treatment [81].

M2-like macrophages exhibit distinct metabolic profiles in comparison to M1-like macrophages. Activation of macrophages with M2-like phenotypic activators, such as IL-4, IL-10, and IL-13, leads to a significant increase in FAO and OXPHOS rates, while glycolysis rates are reduced. These metabolic changes are mediated through the PGC1β signaling pathway, indicating a reliance on lipid metabolism by M2-like macrophages [82–84]. Conversely, FAO is diminished in the M1-like phenotype [85]. Nuclear receptors, including peroxisome proliferator-activated receptor (PPAR) and liver x receptor (LXR), play a regulatory role in lipid metabolism and consequently influence macrophage polarization [76, 86, 87]. Collectively, the metabolic characteristics of M2-like macrophages encompass decreased glycolysis, enhanced FAO, and OXPHOS [77, 78].

Furthermore, it has been demonstrated that Sirtuin 4 (SIRT4) plays a role in lipid metabolism, and the knockdown of SIRT4 leads to an upregulation of FAO genes in TAMs, including PPARδ. Knockdown of SIRT4 results in an elevation of phosphorylated signal transducer and activator of transcription 3 (p-STAT3) protein levels in TAMs, which is essential for the polarization of M2 phenotype. The PPARδ and PPARδ-STAT3 axis actively promotes the polarization of M2-like macrophages. Conversely, inhibition of PPARδ reverses the M2-like polarization induced by SIRT4 knockdown. Additionally, SIRT4 knockdown enhances apoptosis of M1 TAMs, leading to an increase in the M2/M1 ratio. Consequently, the inhibition of SIRT4 in macrophages induces M2-like polarization, augments the M2/M1 ratio through the FAO/PPAR/STAT3 pathway, and ultimately facilitates HCC progression [88]. These findings highlight the potential of targeting fatty acid metabolism in macrophages within the HCC tumor microenvironment as a promising therapeutic strategy.

#### Arachidonic acid metabolism

Arachidonic acid (AA), a polyunsaturated fatty acid, serves as a structural lipid in cellular membranes and acts as a precursor for various bioactive molecules involved in the regulation of biological processes. Among these molecules, prostaglandin E2 (PGE2) plays a crucial role. PGE2 exhibits both pro-tumorigenic and immunosuppressive effects and serves as a key factor in the polarization of macrophages towards an M2-like phenotype [89].

Furthermore, PGE2 induces the upregulation of PD-L1 in macrophages, which subsequently dampens the anti-tumor response of T cells and facilitates tumor growth [90]. Within the TME of HCC, tumor cells produce PGE2, which triggers the polarization of macrophages towards an M2-like phenotype via the cyclic adenosine monophosphate (cAMP) pathway, leading to immune suppression. Conversely, inhibition of PGE2 synthesis can reverse this process and enhance anti-tumor effects [89, 91]. Therefore, targeting agents that modulate PGE2 production holds promise for activating anti-cancer immunity.

### Redox metabolism

The maintenance of redox homeostasis is closely linked to the development of HCC. Notably, studies have demonstrated that the deletion of NADPH oxidase NOX4 triggers the activation of the MYC pathway, resulting in a perturbation of redox homeostasis, perturbed oxidative metabolism, and ultimately facilitating the progression of HCC [92].

As previously mentioned, the polarization of macrophages towards an M2-like phenotype is influenced by intracellular OXPHOS responses, indicating the involvement of redox processes. Suppression of OXPHOS and FAO levels leads to a decrease in M2-like macrophage markers. This can be attributed to the fact that oxidative metabolism fulfills the long-term bioenergetic demands of macrophages during M2 polarization. In contrast, M1 macrophages primarily rely on glycolytic metabolism for rapid energy production. Hence, the regulation of OXPHOS and FAO levels plays a crucial role in macrophage energy utilization, thereby governing M2 polarization [93].

Furthermore, IL-4 significantly enhances the expression of enzymes involved in mitochondrial FAO in macrophages, thereby modulating mitochondrial function and exerting anti-inflammatory effects. The impact of IL-4 on mitochondrial function was confirmed by the reduction in this effect upon the use of mitochondrial inhibitors, further validating the regulatory role of IL-4. STAT6 acts as a transcriptional regulator of the Th2 response in macrophages and coordinates macrophage metabolic programs in response to IL-4. IL-4 triggers STAT6 activation and co-regulates M2 polarization of macrophages [93]. Thus, modulation of oxidative metabolism has the potential to influence the polarization of macrophages. Diminishing the oxidative response of macrophages would result in a decline in their immunosuppressive function, while concurrently enhancing their anti-tumor immune response.

### Lactate metabolism

Cancer cells exhibit a propensity for glycolytic metabolism, wherein they avidly uptake extracellular glucose to generate ATP. Notably, cancer cells exhibit a preference for aerobic glycolysis, commonly known as the Warburg Effect, even in the presence of ample oxygen. This metabolic adaptation, although inefficient in terms of ATP production [94, 95], enables cancer cells to rapidly consume glucose, leading to the accumulation of lactate. Consequently, this metabolic shift impacts immune cell functionality, resulting in the conversion of immune cells into an immunosuppressive phenotype [96].

Excessive accumulation of lactate has been identified as a suppressor of anti-tumor immunity, impeding the proliferation and secretion of cytotoxic cytokines by CTLs. Intracellular acidification resulting from heightened lactic acid consumption by NK cells and T cells has been observed to inhibit IFN-γ production [97]. Meanwhile, lactic acid has the ability to induce the expression of G protein-coupled receptor 132 (Gpr132) in macrophages [16], subsequently promoting the conversion of M1-like macrophages to M2-like macrophages. The ensuing release of trophic factors, metabolic regulators, and immunosuppressive molecules by M2-like macrophages, including vascular endothelial growth factor A (VEGFA), PGE2, IL-6, IL-10, among others, ultimately accelerates tumor progression [12, 95, 98]. Moreover, lactic acid stimulation leads to increased expression of arginase1 (ARG1) and arginase2 (ARG2) in TAMs, thereby facilitating the release of pro-tumorigenic substances and impairing the activity of other immune cells. Consequently, inhibiting Gpr132 can modulate the impact of lactate on macrophage polarization, thereby restraining the metastasis of cancer cells [99].

Furthermore, the pro-tumorigenic impact of macrophages is induced by lactic acid. Targeting the M2-like polarization of macrophages presents a potential strategy to counteract the immunosuppressive effects. Notably, in HCC, macrophages demonstrate elevated expression levels of carbonic anhydrase XII (CA12), which is associated with the survival of M2-like macrophages in an acidic milieu, thereby facilitating HCC progression [100]. Moreover, the production of lactic acid by HCC cells intensifies the acidic microenvironment and exerts significant effects on macrophages.

### Amino acid metabolism

Amino acids play an important role as metabolites closely intertwined with the phenotypic and biological functions of macrophages. A comprehensive summary of amino acid metabolism and its consequential impact on macrophages will be presented.

#### Glutamine metabolism

Glutamine serves as a substrate for various nitrogenous compounds involved in biosynthesis, thereby generating nitrogen for the synthesis of non-essential amino acids and nucleotides. The metabolism of glutamine assumes critical importance in HCC proliferation, as the growth of HCC cells escalates the demand for glutamine and augments its catabolism. This heightened glutamine metabolism represents a significant characteristic of HCC cells [2]. Furthermore, M2 macrophages exhibit elevated levels of glutamine metabolism, glutamine transporter protein, and associated metabolic enzyme expressions [101].

In order to facilitate M2 polarization, TAMs engage in the conversion of glutamate to glutamine. Inhibition of this conversion leads to an upregulation of glycolysis in TAMs, resulting in the reprogramming of macrophages towards a pro-inflammatory M1-like phenotype. Additionally, glutamine enters the TCA cycle through OXPHOS in macrophages, thereby promoting the acquisition of an M2-like polarization phenotype. Notably, restricting glutamine availability has been shown to diminish M2-like polarization [29]. These findings indicate that targeting glutamine metabolism could serve as a potential approach to modulate macrophage phenotype and function.

Furthermore, a previous investigation has demonstrated that fructose stimulation triggers glutamine catabolism, thereby inducing LPS-mediated inflammation and the production of pro-inflammatory cytokines in macrophages [102]. This observation highlights the ability of fructose to induce pro-inflammatory M1-like polarization, thereby enhancing anti-tumor immunity through its impact on glutamine metabolism.

#### Arginine metabolism

Arginine, a metabolite with the ability to undergo catabolism to generate ornithine, serves as a precursor for various biologically significant molecules. For instance, ornithine plays a crucial role in the synthesis of proline and polyamines, which exert an influence on cell division processes [89]. The process of macrophage polarization can be modulated by this metabolic pathway.

Investigations have demonstrated that polyamines contribute to M2-like polarization and inhibit the expression of genes associated with M1-like polarization induced by LPS. Furthermore, the absence of a rate-limiting enzyme involved in polyamine metabolism has been linked to M1-like polarization. As discussed, lactate has been shown to enhance the activity of ARG1 and ARG2 enzymes in TAMs, leading to the catabolism of arginine and the production of compounds that promote cancer cell proliferation. However, it is important to note that both M1 and M2 macrophages utilize arginine, albeit through distinct mechanisms [103].

Moreover, M1-like macrophages employ iNOS to convert arginine into NO. iNOS also upregulates glycolysis to induce M1-like polarization, while concurrently suppressing TCA cycle and OXPHOS processes associated with an M2-like phenotype [29]. Conversely, ARG1 and ARG2 serve as key enzymes utilized by M2-like macrophages to metabolize arginine, thereby attenuating the anti-tumor effects through reduced NO production.

A separate investigation revealed that the knockdown of lncRNA cox-2 resulted in a reduction of iNOS and TNF-α levels in M1-like macrophages. This reduction in iNOS and TNF-α levels led to the suppression of the macrophages’ ability to inhibit the proliferation of HCC cells and promote apoptosis. Conversely, the knockdown of lncRNA cox-2 increased the expression of ARG1 and IL-10 in M2-like macrophages. This increase in ARG1 and IL-10 expression promoted the proliferation of HCC cells and inhibited apoptosis in M2-like macrophages, respectively [104]. These findings provide evidence that the knockdown of lncRNA cox-2 can weaken the anti-tumor immune response by modulating macrophage polarization in HCC. The underlying molecular mechanism involves the differential impact of lncRNA cox-2 on iNOS and ARG1. Consequently, altering the utilization of arginine by macrophages to specifically induce iNOS or inhibit ARG1 and ARG2 may have implications for macrophage phenotypes.

#### Tryptophan metabolism

The involvement of tryptophan metabolism in HCC cells is of significant importance. Specifically, the presence of tryptophan 2, 3-dioxygenase (TDO2) in HCC cells has been linked to malignant characteristics in patients with HCC. TDO2 facilitates the metabolic conversion of tryptophan, resulting in the production of kynurenine along the kynurenine (Kyn) pathway. The activation of the aryl hydrocarbon receptor (AhR) by kynurenine plays a regulatory role in the growth and invasion of HCC [105].

Moreover, indoleamine 2, 3-dioxygenase (IDO) can oxidize tryptophan in TAMs, leading to a reduction in tryptophan availability for T cells and the generation of metabolites that suppress T cell functions [89]. The restoration of T cell function through the use of IDO inhibitors further supports the close association between tryptophan and M2-like polarization [29]. These findings provide additional evidence for the contribution of tryptophan metabolism to macrophage polarization and function in HCC.

Furthermore, it has been observed that the metabolite of tryptophan, nicotinamide adenine nucleotide (NAD), has the ability to influence the levels of the inflammatory cytokine TNF-α in macrophages [106]. Obviously, an elevated NAD level is associated with an up-regulation of TNF-α, which is indicative of M1-like polarization. These findings collectively suggest that the utilization of tryptophan by macrophages holds promise for exerting anti-tumor effects. Consequently, an increase in NAD levels or a decrease in IDO activity would likely support an anti-tumor response.

### Purine metabolism

Purine, a crucial metabolite involved in energy production, metabolic regulation, and various other physiological processes, undergoes conversion into uric acid for elimination. Notably, investigations have revealed a substantial activation of purine metabolism and heightened activity within the purine biosynthesis pathway in patients with HCC [107]. Furthermore, the upregulation of genes associated with the purine metabolism pathway has been observed, and this dysregulation has been linked to an unfavorable prognosis in HCC patients [108].

Purine metabolism exhibits a close association with the biological functionality of macrophages, particularly in the context of TAMs. Studies have demonstrated a direct correlation between heightened purine metabolism in TAMs and compromised efficacy of immunotherapeutic interventions, as well as the promotion of pro-tumorigenic activities. The augmented purine metabolism in TAMs is characterized by a diminished capacity for antigen presentation, resulting in the suppression of M1-like macrophages. This suppression is attributed to the downregulation of antigen presentation-related genes in macrophages with elevated purine metabolism. Remarkably, the expression levels of purine metabolism genes exhibit an inverse relationship with those of antigen-presenting genes [109].

Furthermore, the immunosuppressive molecule TREM2, expressed in macrophages, displays a positive correlation with the purine metabolism score. Macrophages expressing TREM2 exhibit reduced levels of antigen presentation-related proteins, such as MHC-II (I-A/I-E) and MHC-I (H-2D), indicative of impaired antigen presentation capabilities. These findings further underscore the negative regulatory role of purine metabolism in macrophage antigen presentation [109]. Meanwhile, heightened purine metabolism confers enhanced immunosuppressive and angiogenic capacities, thereby bolstering the functions associated with M2-like macrophages. Thus, the intricate interplay between purine metabolism and M2-like polarization emerges as a promising avenue for harnessing the anti-tumor potential.

### Acid–base metabolism

The maintenance of acid–base homeostasis is of paramount importance for cellular survival, as cells necessitate an environment with optimal pH levels. As previously discussed, the presence of lactic acid contributes to the establishment of an acidic milieu that fosters tumor progression. Perturbations in pH levels exert a profound influence on the biological functionality of macrophages.

Emerging evidence highlights a close correlation between the pH value and the phenotype and function of macrophages. For instance, the administration of chloroquine (CQ) has been shown to elevate the pH level within macrophage lysosomes, thereby eliciting M1-like polarization through calcium-mediated mechanisms. Furthermore, CQ induces a metabolic shift in macrophages from OXPHOS to glycolysis [110, 111]. Moreover, the manipulation of hydrogen binding and removal within the microenvironment, facilitated by specific agents like calcium carbonate (CaCO3), promotes an alkaline milieu that favors M1-like polarization [112].

In addition, TAMs have the ability to adapt and survive within the acidic TME of HCC through the production of vacuolar-type ATPase (V-ATPase). Notably, the inhibition of V-ATPase has been demonstrated to induce a shift in macrophage polarization from M2-like to M1-like, accompanied by an upregulation of pro-inflammatory cytokine expression, such as TNF-α [113]. These findings collectively underscore the impact of acid–base balance on macrophage polarization. M1-like macrophages exhibit a preference for an alkaline milieu, while M2-like phenotypes display an affinity for acidic conditions. Consequently, an elevation in pH levels can prompt macrophages to transition towards an M1-like phenotype, thereby fostering an environment conducive to anti-tumor immune responses.

### Iron metabolism

Inorganic elements play a crucial role in maintaining cellular functions, with iron (Fe) being intricately linked to macrophage polarization. The distinct iron utilization patterns of M1 and M2 macrophages contribute to their respective functions. M2 macrophages are involved in tissue healing and employ scavenger receptors like CD163 to uptake and process heme, thereby generating iron and producing anti-inflammatory factors. Conversely, iron release supports tumor progression by promoting tissue repair, cellular proliferation, and immune modulation. In contrast, M1-type macrophages primarily uptake and store iron, thereby attenuating the immunosuppressive effects associated with iron release [103, 114].

As mentioned, HCC cells generate lactate, which induces M2-like polarization. However, the introduction of ferric citrate has been shown to suppress lactate-mediated M2-like polarization [115]. Therefore, increased iron uptake by macrophages facilitates M1-like polarization, thereby enhancing anti-tumor immunity.

## Treatment prospects in targeting macrophage metabolism

Numerous strategies have been explored to effectively target TAMs in HCC. One promising immunotherapeutic approach involves the augmentation of M1-like polarization, which holds potential for the treatment of HCC (Fig. 1). Additionally, the modulation of metabolic molecules and associated enzymes represents another avenue to regulate the pro-inflammatory and anti-inflammatory functions of macrophages, thereby facilitating an anti-tumor response. Subsequently, we will delve into therapeutic interventions that target macrophage metabolism pathways, providing a comprehensive analysis of their potential implications in HCC treatment.

### ATO combined with CTS therapy for HCC

**Fig. 4:**
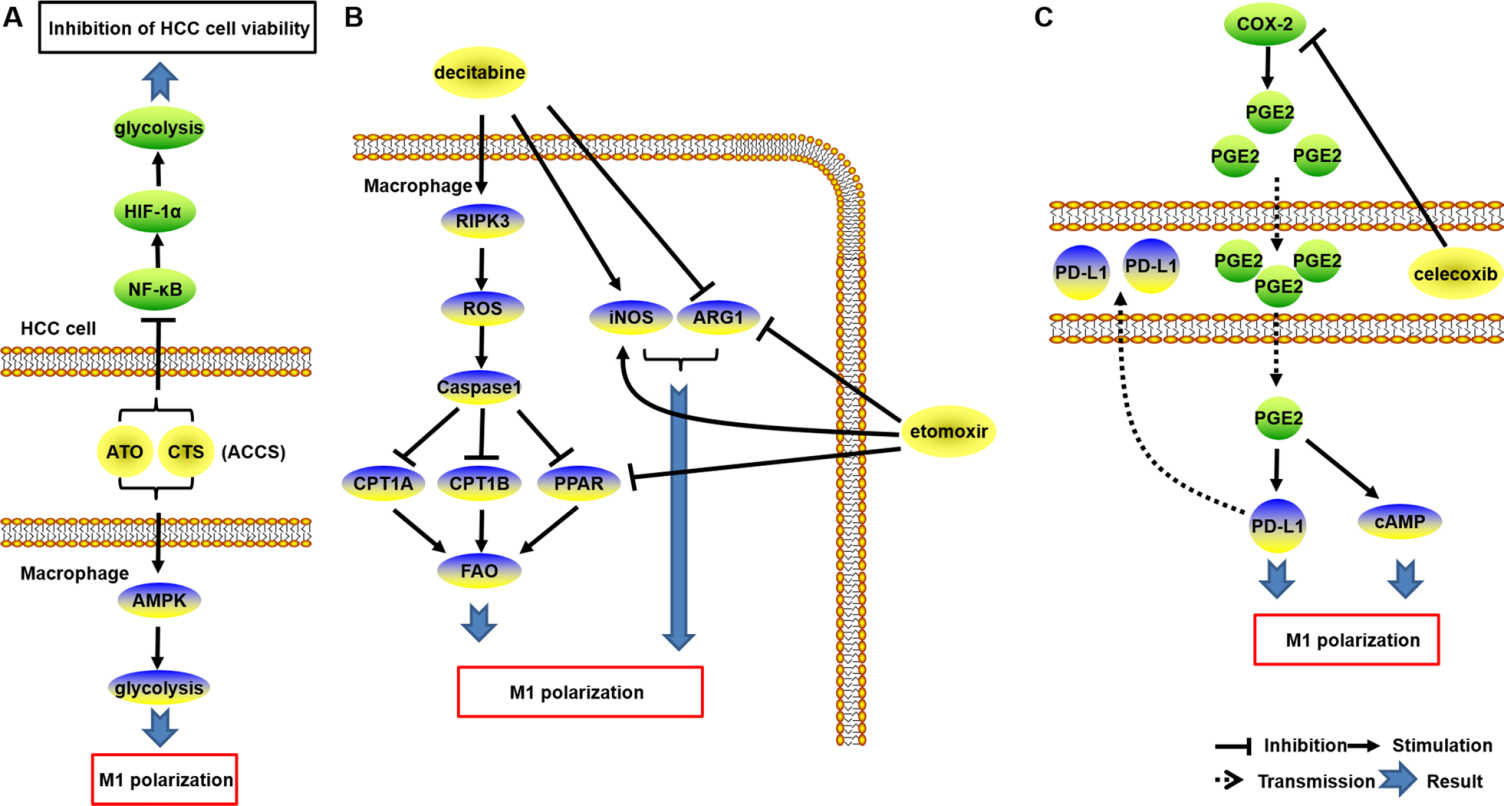
The mechanisms of therapeutics for HCC through regulating macrophage metabolism. **A** ACCS modulates glycolysis metabolism, leading to the induction of M1-like polarization in macrophages. This metabolic shift not only suppresses HCC cell survival but also exerts potent anti-tumor effects. **B** The combination of decitabine and etomoxir acts on macrophages by regulating FAO and amino acid metabolism, thereby promoting M1-like polarization. This synergistic effect contributes to the therapeutic potential against HCC. **C** Celecoxib, through the inhibition of COX-2-related PGE2 production, facilitates the promotion of M1-like polarization in macrophages

Arsenic trioxide (ATO) has emerged as a primary therapeutic option for acute promyelocytic leukemia (APL). Meanwhile, Cryptotanshinone (CTS), an extract derived from the traditional Chinese medicine Salvia miltiorrhiza, has gained attention for its potential in HCC treatment. Notably, the combination of ATO and CTS (ACCS) has been employed in HCC therapy. In a recent study, it was demonstrated that ACCS administration led to an elevation in AMPK phosphorylation, thereby activating the AMPK pathway. This activation, in turn, induced glycolysis in macrophages and promoted M1-like macrophage polarization. Furthermore, ACCS exhibited the ability to suppress glycolysis in HCC cells by inhibiting the NF-κB/HIF-1α pathway [116] (Fig. 4A). Consequently, ACCS represents a promising therapeutic approach for HCC, as it effectively targets both macrophages and cancer cells.

### Decitabine combined with etomoxir therapy for HCC

Receptor-interacting protein kinase 3 (RIPK3) is a serine/threonine kinase that plays a crucial role in activating immunological responses and facilitating necrosis [117]. RIPK3 expression was found to be down-regulated in TAMs within HCC. This down-regulation of RIPK3 was observed to stimulate the PPAR pathway, thereby inducing fatty acid metabolism through the suppression of the ROS-Caspase-1 pathway. The latter pathway is known to be associated with the immunological function of TAMs and inhibits HCC progression [118, 119].

In Phase II clinical trials (NCT02264873), decitabine, a therapeutic agent, has been demonstrated to effectively inhibit myelodysplastic syndrome, liver metastasis, and tumor cell proliferation. Decitabine treatment was found to enhance RIPK3 expression in TAMs while suppressing PPAR expression. This suppression of PPAR prevented FAO by reducing the levels of carnitine palmitoyltransferase 1 A (CPT1A) and CPT1B, which are rate-limiting enzymes in fatty acid metabolism. Furthermore, decitabine treatment resulted in decreased expression of ARG1, a marker associated with M2-like TAMs, and increased expression of iNOS, a marker associated with M1-like TAMs. These findings confirmed that decitabine treatment prevented M2 polarization of TAMs and promoted M1 polarization through the modulation of RIPK3. Consequently, decitabine effectively reversed the pro-tumoral effects induced by RIPK3 deficiency in HCC [118, 119].

Furthermore, it has been demonstrated that the inhibition of CPT1 by etomoxir resulted in a decrease in ARG1 expression. This decrease in ARG1 expression subsequently interfered with the expression of PPARA and PPARG, thereby impacting FAO metabolism. In parallel, etomoxir treatment upregulated the expression of iNOS in TAMs, ultimately facilitating an anti-tumor effect. Consequently, the combination therapy of decitabine with etomoxir holds promise in controlling fatty acid metabolism, leading to the inflammatory polarization of TAMs, as depicted in Fig. 4B [118, 119].

### Celecoxib regulates macrophage metabolism and function in tumors

The abundant expression of cyclooxygenase-2 (COX-2) in various cancers, including HCC, is closely associated with inflammation, which plays a crucial role in the initiation and progression of HCC and is indicative of a poor prognosis in patients. COX-2 also facilitates the production of PGE2, thereby exerting pro-tumoral effects. Consequently, the potential anti-cancer function of COX-2 knockdown has been identified [120, 121].

Furthermore, the elevated expression of COX-2 in HCC cells has been experimentally confirmed to induce M2-like polarization, leading to the suppression of T cell cytotoxicity. The administration of celecoxib, a COX-2 inhibitor, effectively inhibits PGE2 formation [122], consequently reducing the presence of M2-like TAMs as depicted in Fig. 4C. Simultaneously, celecoxib enhances the anti-tumor function by augmenting T cell cytotoxicity [123]. Therefore, the targeting of COX-2/PGE2-based metabolic molecules holds promise in improving immunotherapy outcomes for HCC.

### Other therapies for regulating macrophage metabolism and function in tumor

The additional studies that have explored the use of macrophage metabolism-targeting drugs for tumor therapy are summarized in Table 1.

**Table 1:** Drugs that target metabolism in macrophages

Drug	Mechanism	References
Glucose metabolism		
Insulin	Promoting glycolysis in monocyte-phagocytes to enhance phagocytosis on tumor cells	[124]
β-Glucan	Promoting glycolysis in macrophages and thereby inducing M1-like polarization	[125]
Fucoidan	Inhibiting the oxidation reaction of macrophages, promoting glycolysis, inducing M1 polarization, and playing an anti-tumor effect	[126]
Ibrutinib	Inhibiting glycolysis in monocyte-phagocyte and weakening its phagocytosis on tumor cells	[124]
Lipid metabolism		
Simvastatin	Consuming lipids and transforming M2-like macrophages to M1-like	[127]
Rapamycin + Hydroxychloroquine	Disrupting FAO in macrophages to inhibit M2-like polarization	[128]
Perhexiline	Inhibiting oxidative phosphorylation and fatty acid metabolism to promote M1-like polarization	[129]
Metformin	Inhibiting FAO to induce macrophage polarization to M1 phenotype, suppressing anti-inflammatory macrophage infiltration through decreasing COX2 and PGE2	[29, 130]
Indomethacin	Inhibiting COX and PGE2, inducing anti-tumor effect by macrophages	[131]
Isoliquiritigenin	Inhibition of PGE2 production and reduction of M2-like polarization	[132]
Salvia miltiorrhiza Bunge aqueous extract	Inhibiting COX-2, reducing PGE2 production, decreasing tumor-promoting macrophage infiltration, and mediating anti-tumor immune responses	[133]
Fe-5,5′-azosalicylic acid nanoscale coordination polymer nanomedicines	Producing 5-aminosalicylic acid to reduce COX-2 and PGE2 expression, conversely, generating Fe3 + to induce M1-like polarization	[134]
5-Aminolevulinic Acid	Inhibiting COX-2 and PGE2 expressions, suppressing tumor by macrophages	[135]
Lactic acid metabolism		
3-Bromopyruvate	Inhibiting tumor-promoting macrophages by decreasing lactate production	[136]
Albiziabioside A + Dichloroacetate acid	Inhibiting lactate accumulation to reduce M2 macrophages and reprogram anti-tumor microenvironment	[137]
Dual PI3Kδ/γ Inhibitor RP6530	Reducing lactate, inducing M1-like polarization and inhibiting tumor progression	[138]
Amino acid metabolism		
6-Diazo-5-oxo-l-norleucine	Inhibiting glutamine metabolism, suppressing IDO expression, and inducing pro-inflammatory macrophages	[139]
6-Gingerol	Inhibiting ARG expression, promoting iNOS and NO expression, enhancing M1-like polarization, and exerting anti-tumor effect	[140]
Triptolide	Reducing ARG1 expression and decreasing M2-like polarization	[141]
1,3-Diaryl-pyrazin-6-one-5-carboxamides	Inhibiting IDO level and reducing immunosuppressive macrophage infiltration	[142]
Sulfasalazine	Inhibiting cystine-glutamate exchange (xCT) and thereby inducing M2-like polarization	[143]
Acid–base metabolism		
Anti‐V‐ATPase‐V0a2 antibody	Inhibiting proton pump activity to induce M1-like macrophage	[144]
Pantoprazole	Inhibiting proton pump to induce M1-like polarization and activating anti-tumor immunity	[145]
Iron metabolism		
Iron oxide nanoparticles	Iron absorbed by macrophages and thereby replenished to promote M1-like polarization	[146]
Iron chelated melanin-like nanoparticles	Iron supplemented by macrophages to induce M1-like polarization	[147]
Intracellular iron chelator (TC3-S)2	Transforming macrophage to iron-absorbing M1-like phenotype to play anti-tumor effects	[148]

## Conclusion and perspective

It is well-established that cellular metabolism plays a crucial role in determining cell function and the TME in HCC. The TME, in turn, can influence the metabolism and polarization of macrophages, thereby impacting their phenotype and function. Notably, these altered macrophages play a pivotal role in promoting the carcinogenesis and progression of HCC, as outlined in Table 2. Consequently, targeting M2-like TAMs and their associated metabolism holds significant potential in enhancing the efficacy of anti-tumor therapy, with several studies confirming its clinical value. Therefore, it is imperative to continue investigating the intricate relationship between metabolism and the resulting alterations in the phenotype and function of TAMs. Such investigations may pave the way for the development of novel strategies aimed at promoting anti-tumor immunity in HCC.

**Table 2:** The relationship between macrophage and HCC involved pathway and clinical outcome

Cell	Pathway	Clinical outcome	References
The effect of HCC cells on macrophages			
HCC cells	FOXO1/ IRF1/ NO axis	Reprogramming macrophages and inhibiting HCC progression	[35]
HCC cells	PKC/ZFP64/CSF1 axis	Inducing M2-like polarization to mediate immune suppression and resistance to anti-PD1 therapy	[42]
HCC cells	MiR4458HG/ARG1 axis	Promoting M2-like polarization and creating an environment conducive to HCC cells	[73]
HCC cells	PGE2/PD-L1; PGE2/cAMP pathway	Reducing anti-tumor response of T cells, promoting M2-like polarization and inducing HCC growth	[89–91]
HCC cells	Lactic acid/Gpr132; Lactic acid/ARG1, ARG2 axis	Transforming M1-like macrophages into M2-like; Promoting the release of tumor substances, damaging the activity of other immune cells, and enhancing the metastasis of cancer cells	[12, 95, 98, 99]
The effect of macrophages on HCC			
Macrophages	TREM1/PI3K/AKT/mTOR pathway down-regulation	Transforming M2-like macrophages into M1-like, preventing HCC cell invasion and metastasis	[43]
Macrophages	TNFR1/NF- κ B/p38/MAPK pathway	Reducing immunosuppressive effect of TAMs, promoting T cell related cytotoxicity, and inducing HCC cell apoptosis	[71]
Macrophage	Wnt2b/β/C-Myc pathway	Promoting the conversion of M2-like TAMs and enhancing HCC progression	[72]
Macrophage	Nogo-B-Yap/Taz axis	Promoting M2-like TAMs polarization and HCC cells proliferation	[74]
Macrophage	LPS/HIF1 α/mTOR axis	Promoting glycolytic activity, leading to M1-like phenotype polarization, and inhibiting tumor progression	[76]
Macrophage	SIRT4/FAO/PPAR/STAT3 pathway	Promoting M2-like polarization and HCC progression	[88]
Macrophage	Glutamic acid/glutamine/OXPHOS pathway	Promoting M2-like polarization and tumor progression	[29]
Macrophage	Arginine/iNOS/NO, glycolysis pathway	Promoting M1-like polarization and inhibiting tumor progression	[29]
Macrophage	LncRNA cox-2/iNOS, TNF- α axis	Inhibiting HCC cell proliferation and promoting cell apoptosis	[104]
Macrophage	Tryptophan/IDO pathway	Reducing tryptophan used by T cells, producing metabolites to inhibit T cell function, and inhibiting anti-tumor immunity	[89]

## Data Availability

Not applicable.

## Abbreviations

ACCS: ATO Joint CTS
AhR: Aryl hydrocarbon receptor
APL: Acute promyelocytic leukemia
ARG1: Arginase 1
ARG2: Arginase 2
ATO: Arsenic trioxide
ATP: Adenosine 5′-triphosphate
CA12: Carbonic anhydrase XII
cAMP: Cyclic adenosine monophosphate
CD163: Scavenger receptor cysteine-rich type 1 protein M130
CD206: Mannose receptor C-type 1
CD244: Natural killer cell receptor 2B4
CD48: Signaling lymphocytic activation molecule 2
CD80: T-lymphocyte activation antigen CD80
CD86: T-lymphocyte activation antigen CD86
CKI: Cmpound kushen injection
c-Myc: MYC proto-oncogene
BHLH: Transcription Factor
COX-2: Cyclooxygenase-2
CPT1A: Carnitine palmitoyltransferase 1 A
CQ: Chloroquine
CSF1: Colony-stimulating factor 1
CTLs: Cytotoxic T lymphocytes
CTS: Cryptotanshinone
FA: Fatty acids
FAO: Fatty acid oxidation
FOXO1: Forkhead box protein O1
GAPDH: Glyceraldehyde-3-phosphate dehydrogenase
HBV: Hepatitis B virus
HCC: Hepatocellular carcinoma
HCV: Hepatitis C virus
ICIs: Immune checkpoint inhibitors
IDO: Indoleamine 2,3 dioxygenase
IFN-γ: Interferon-γ
IL-10: Interleukin 10
IL-12: Interleukin 12
IL-13: Interleukin 13
IL-4: Interleukin 4
iNOS: Inducible nitric oxide synthase
IRF1: Interferon regulatory factor 1
Kyn: Kynurenine
LPS: Lipopolysaccharide
LXR: Liver x receptor
MAPK: Mitogen-activated protein kinase
mTOR: Mechanistic target of rapamycin
NAD: Nicotinamide adenine nucleotide
NAFLD: Non-alcoholic fatty liver disease
NASH: Non-alcoholic steatohepatitis
NF-κB: Nuclear factor-κB
NK cell: Natural killer cell
NO: Nitric oxide
Nogo-B: Reticulon 4B
OXPHOS: Oxidative phosphorylation
p38: Tumor necrosis factor receptor superfamily member 1
PD-1: Programmed cell death 1
PD-L1: Programmed death ligand 1
PGE2: Prostaglandin E2
PKCα: Protein kinase C alpha
PPAR: Proliferator-activated receptor
PPP: Pentose phosphate pathway
PS: Phosphatidylserine
p-STAT3: Phosphorylated signal transducer and activator of transcription 3
RIPK3: Receptor-interacting protein kinase 3
ROS: Reactive oxygen species
SIRT4: Sirtuin 4
SPON2: Spondin 2
TAMs: Tumor-associated macrophages
Taz: Tafazzin
TCA cycle: Tricarboxylic acid cycle
TDO2: Tryptophan 2,3-dioxygenase
TGF-β: Transforming growth factor beta
TKIs: Tyrosine kinase inhibitors
TLR: Toll-like receptor
TLR9: Toll-like receptor 9
TME: Tumor microenvironment
TNFR1: Tumor necrosis factor receptor superfamily member 1
Treg: Regulatory T cells
TREM1: Triggering receptor expressed on myeloid cells 1
VEGFA: Vascular endothelial growth factor A
Wnt2b: Wnt family member 2B
Yap: Yes-associated protein
ZFP64: Zinc finger protein 64
